# A Strategy to Modulate the Bending Coupled Microwave Magnetism in Nanoscale Epitaxial Lithium Ferrite for Flexible Spintronic Devices

**DOI:** 10.1002/advs.201800855

**Published:** 2018-11-06

**Authors:** Lvkang Shen, Guohua Lan, Lu Lu, Chunrui Ma, Cuimei Cao, Changjun Jiang, Huarui Fu, Caiyin You, Xiaoli Lu, Yaodong Yang, Lang Chen, Ming Liu, Chun‐Lin Jia

**Affiliations:** ^1^ School of Electronic and Information Engineering and State Key Laboratory for Mechanical Behavior of Materials Xi'an Jiaotong University Xi'an 710049 China; ^2^ School of Material Science and Engineering and State Key Laboratory for Mechanical Behavior of Materials Xi'an Jiaotong University Xi'an 710049 China; ^3^ School of Physics Science and Technology Lanzhou University Lanzhou 730000 China; ^4^ School of Material Science and Engineering Xi'an University of Technology Xi'an 710048 China; ^5^ State Key Discipline Laboratory of Wide Band Gap Semiconductor Technology School of Microelectronics Xidian University Xi'an 710071 China; ^6^ Frontier Institute of Science and Technology Xi'an Jiaotong University Xi'an 710049 China; ^7^ School of Physics Southern University of Science and Technology Shenzhen 518055 China; ^8^ Ernst Ruska Centre for Microscopy and Spectroscopy with Electrons Forschungszentrum Jülich D‐52425 Jülich Germany

**Keywords:** epitaxial oxide thin films, ferromagnetic resonance, flexible devices, magnetism

## Abstract

With the development of flexible electronics, the mechanical flexibility of functional materials is becoming one of the most important factors that needs to be considered in materials selection. Recently, flexible epitaxial nanoscale magnetic materials have attracted increasing attention for flexible spintronics. However, the knowledge of the bending coupled dynamic magnetic properties is poor when integrating the materials in flexible devices, which calls for further quantitative analysis. Herein, a series of epitaxial LiFe_5_O_8_ (LFO) nanostructures are produced as research models, whose dynamic magnetic properties are characterized by ferromagnetic resonance (FMR) measurements. LFO films with different crystalline orientations are discussed to determine the influence from magnetocrystalline anisotropy. Moreover, LFO nanopillar arrays are grown on flexible substrates to reveal the contribution from the nanoscale morphology. It reveals that the bending tunability of the FMR spectra highly depends on the demagnetization field energy of the sample, which is decided by the magnetism and the shape factor in the nanostructure. Following this result, LFO film with high bending tunability of microwave magnetic properties, and LFO nanopillar arrays with stable properties under bending are obtained. This work shows guiding significances for the design of future flexible tunable/stable microwave magnetic devices.

Over the past decades, the progress in the development of flexible functional materials has promoted the application of traditional electronics in a wide range, such as flexible transparent display,^1^, ^2^, ^3^ wearable memory devices,^4^, ^5^, ^6^ multifunctional sensors,^7^, ^8^, ^9^, ^10^ and etc. To meet the practical application requirements of flexible devices, besides the physical characteristics, the mechanical flexibility of functional materials gradually plays an important role in the materials selection.^11^ For example, the change of resistance during repeated bending cycles is one of the most important benchmarks for the flexibility of soft electrodes. For flexible spintronics based applications, magnetic materials with high spin polarization as well as excellent mechanical flexibility are highly desirable to meet the requirement of high performance and stability.^12^, ^13^, ^14^, ^15^, ^16^ In the previous studies, bending‐deformation‐related magnetic hysteresis (M‐H) loops have been found in several flexible epitaxial oxide thin films with high crystalline quality, such as CoFe_2_O_4_,^17^, ^18^ CuFe_2_O_4_,^19^ LiFe_5_O_8_,^20^ Fe_3_O_4_,^21^ etc. The results indicate that the effects of bending‐induced mechanical strains on the magnetic properties are different for different materials. To meet the future demands in precise control and design of flexible spintronic systems,^19^, ^21^ it is of significant importance to establish the relationships between magnetic properties and complex mechanical motions. However, the knowledge of the bending coupled dynamic magnetic properties is still poor when integrating materials in flexible devices. Specifically, magnetocrystalline anisotropy, demagnetization field, and magnetostriction are often considered to play the key role in the coupling between bending and dynamic magnetism, but there is still lack of quantitative analysis for the specific contribution from the three factors.^22^, ^23^, ^24^

As one of the experimental techniques with high sensitivity, ferromagnetic resonance (FMR) is an effective tool for studying static and dynamic characteristics of magnetic thin films.^25^, ^26^ It can be employed to correlate the magnetic anisotropy (MA) to the structure of the sample. According to the report by Liu et al., bending deformation could effectively modulate the out‐of‐plane (OOP) FMR resonance field (*H*_r_) in the (111)‐oriented CuFe_2_O_4_ thin films on flexible fluorophlogopite mica (F‐Mica) substrate, which provides opportunities for wearable mechanics‐magnetic sensors and flexible microwave signal processing devices.^19^ Nevertheless, the complex in‐plane (IP) multidomain structure on F‐Mica substrate and strain‐sensitive magnetic properties of CuFe_2_O_4_ make it difficult to distinguish the contribution of the demagnetization field from that of the magnetocrystalline anisotropy. In addition, nanoscale materials with stable microwave dynamic magnetic properties under bending are highly required in the application of the next‐generation flexible spintronic devices.^16^, ^27^ For these requirements, a model sample with simple crystal structure is expected to allow further establishing the precious relationship between bending deformation and the tested FMR signals. Among various microwave ferrites, LiFe_5_O_8_ (LFO) is one of the promising candidates owing to its low loss at high microwave frequencies coupled with excellent magnetic properties.^28^, ^29^ In addition, LFO films have been reported to besuccessfully transferred onto flexible substrates by the wet etching and transfer methods.^16^ Relative stable M‐H loops under bending were reported for the transferred LFO films, which was explained by the relatively low magnetostrictive coefficient.^20^ Hence, it is expected that the flexible LFO films are reproducible with different OOP crystal orientations to investigate the origin of the bending tuned microwave magnetism in flexible magnetic thin films. Besides, the influence from the demagnetization field can be discussed by tuning the nanostructure of the LFO film, since it was reported that nanostructure could effectively modulate the demagnetization factor of materials.^30^, ^31^ In the former work, (111)‐oriented CoFe_2_O_4_ nanopillar arrays have been successfully grown on flexible fluorophlogopite substrates by the physical deposition and wet etching process, which can be further extended to form the LFO nanostructures.^22^

In this work, epitaxial LFO thin films (≈150 nm) with (001), (110), (111) OOP orientations have been successfully transferred on flexible polydimethylsiloxane (PDMS) substrates (see Experimental Section and Figure S1, Supporting Information), as shown in the inset of **Figure** **1**a–c. In addition, (111)‐oriented LFO nanopillar arrays were fabricated on flexible F‐Mica substrates. Angular‐dependent FMR measurements were conducted for all samples under released or bending states. Interestingly, it was shown that the variation of the main resonance field for the LFO samples under bending highly depends on the MA property of the samples under nonbending. In other words, the shape deformation induced easy axis deviation from its initial position is mainly responsible for the bending modulated FMR spectra in the flexible LFO samples, which can be roughly predicted by analyzing the MA energy of the sample with unbent state. This work provides a fundamental basis for the design of future FMR bending sensors or bending tunable/stable spintronic devices.

**Figure 1:**
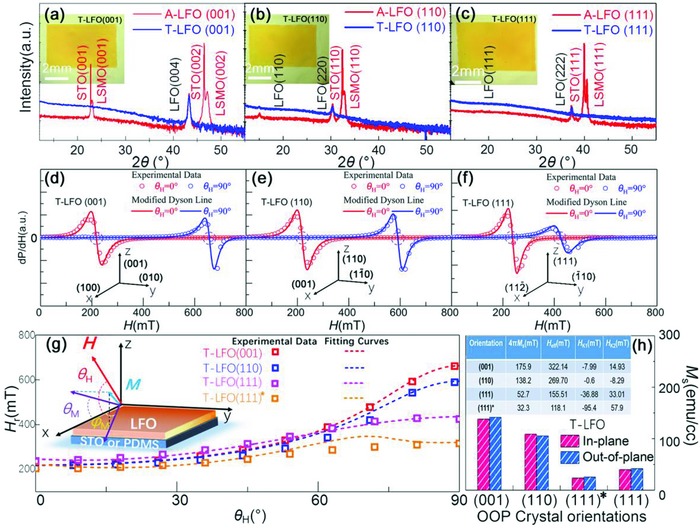
XRD θ–2θ scans of A‐LFO and T‐LFO films with a) (001), b) (110), and c) (111) OOP orientations, respectively. Inset of (a)–(c): corresponding photographs of the T‐LFO films. FMR spectra of d) T‐LFO (001), e) T‐LFO (110), and f) T‐LFO (111) along IP (θ_H_ = 0°) and OOP (θ_H_ = 90°) orientations. g) Experimental (scatter) and fitting curves (dash line) of angular θ_H_‐dependent *H*_r_ for T‐LFO films with different orientations. Upper left corner of (g): schematic illustration of FMR spectroscopy experimental set‐up for the film sample. h) *M*_s_ of T‐LFO films with different OOP orientations. Inset of (h): a table listed the 4*πM*_s_ value and the fitting parameters *H*_eff_, *H_K_*_1_, and *H_K_*_2_.

To explore the crystal structure evolution during the etching and transfer process, typical X‐ray diffraction (XRD) θ–2θ scans were performed on the transferred LFO (T‐LFO) thin films and the as‐grown LFO/La_0.67_Sr_0.33_MnO_3_ (LSMO) films on SrTiO_3_ (STO) substrates (A‐LFO) with different crystal orientations. As shown in Figure 1a–c, all of the LFO films show excellent single orientation without any detectable peaks of impurity phases. After the wet etching and transfer process, all of the peaks from the STO substrate and the LSMO buffer layer disappeared, only leaving the peaks from the transferred LFO phases. Compared with the as‐grown film, only tiny changes of interplanar spacing (<0.1%) along OOP directions is found in the T‐LFO films, which are in the range of the test error. The lattice spacing of LFO (004) plane, LFO (110) plane, and LFO (111) plane are 2.08, 5.89, and 4.81 Å, respectively, revealing that all of the transferred films are fully relaxed. ϕ scans were conducted to confirm the IP crystal structural relation of the LFO films, as shown in Figure S2 in the Supporting Information. The azimuthal scan of the LFO layers with both (001) and (110) OOP orientations shows the same symmetry with that of the STO substrate, revealing typical single‐domain feature with the rotation pole along [001] and [110] in cubic, respectively. For the films on STO (111) substrates, the LFO layers exhibit sixfold symmetry, indicating a multidomain structure. Although we have not obtained the ϕ scan result for T‐LFO (111) dueto the low signal‐to‐noise ratio (SNR), according to the results for T‐LFO (001) and T‐LFO (110) films, it could be concluded that this transfer method retains the crystal symmetry of the as‐grown film.

FMR measurements were conducted on the T‐LFO films. As shown in the upper left corner of Figure 1g, ***H*** represents the external magnetic field, which is set in the *xz*‐plane with an azimuthal angle θ_H_. ***M*** is the effective magnetization. θ_M_ and ϕ_M_ represent the azimuthal angles of ***M*** in the *xz*‐plane and in the *xy*‐plane, respectively. In the experiment, angular θ_H_‐dependent FMR signals were measured by rotating ***H*** in the *xz*‐plane. The bottom of Figure 1d–f shows the relationships between the crystallographic orientations of the LFO films and the *x*, *y*, and *z* axes. As there exists an asymmetry in the experimental FMR lines due to the dispersion components of the susceptibility of the microwave electromagnetic field, modified Dyson function is applied to fit the experimental FMR lines,^32^, ^33^ as shown in Figure 1d–f. From this fitting, the *H*_r_ can be obtained with better accuracy. To understand the origin of the MA properties of the T‐LFO films with different crystallographic orientations, formula fitting method is applied based on the experimental results of the θ_H_‐dependent *H*_r_. Considering the contributions of the crystal orientations and specimen shape, the free energy related to the magnetization of the films can be expressed as^34^

(1) $$\begin{array}{l} F=-HM_{s}\left[\cos\theta_{M}\cos\theta_{H}\cos(\varphi_{M}-\varphi_{H})+\sin\theta_{M}\sin\theta_{H}\right]\\ \;\;\;\;\;\;+2\pi NM_{s}^{2}\sin^{2}\theta_{M}-K_{u}\sin^{2}\theta_{M}+F_{\mathrm{MAE}}+F_{\mathrm{ME}} \end{array}$$

The first term is the Zeeman energy. The second one is the demagnetization field energy characterized by the saturation magnetization *M*_s_ and the demagnetization factor *N* (*N* = *N*_z_ = 1 for thin film). The third and fourth terms represent the OOP uniaxial anisotropy energy characterized by a constant *K*_u_ and the magnetocrystalline anisotropy energies *F*_MAE_ characterized by constants *K_i_* (*i* = 1,2,3…), respectively. The last term is the magnetoelastic energy, which is discussed by the orthogonal normal stresses model^35^(see more details in the Supporting Information). In this study, the T‐LFO films are fully relaxed so that the *F*_MAE_ can be ignored. Hence, based on the Smit–Beljers formalism,^36^ while neglecting the contribution from constant *K_i_* (*i* > 2) and setting ϕ_M_ = ϕ_H_ = 0, the expression for the FMR frequency can be derived as

(2) $$\begin{array}{l} {\left(\frac{\omega }{\gamma }\right)}^{2}=[H_{r}\cos\left(\theta_{M}-\theta_{H}\right)+H_{\mathrm{eff}}\cos\left(2\theta_{M}\right)+A_{1}\left(\theta_{M}\right)H_{K1}+\\ \;\;\;\;\;\;\;\;\;\;\;\;\;A_{2}\left(\theta_{M}\right)H_{K2}]\times \;[H_{r}\cos\left(\theta_{M}-\theta_{H}\right)-H_{\mathrm{eff}}\sin^{2}\theta_{M}+\\ \;\;\;\;\;\;\;\;\;\;\;\;\;B_{1}(\theta_{M})H_{K1}+B_{2}\left(\theta_{M}\right)H_{K2}] \end{array}$$

(3) $$\begin{array}{l} \frac{1}{M_{s}}\;\frac{\partial F}{\partial \theta_{M}}=H_{r}\sin\left(\theta_{M}-\theta_{H}\right)+\frac{1}{2}H_{\mathrm{eff}}\sin\left(2\theta_{M}\right)+\\ \;\;\;\;\;\;\;\;\;\;\;\;\;\;\;\;C_{1}\left(\theta_{M}\right)H_{K1}+C_{2}\left(\theta_{M}\right)\;H_{K2}=\;0 \end{array}$$

where ω is the microwave angular frequency, γ is the gyromagnetic factor (≈2.768 GHz kOe^−1^). *H*_eff_ represents the effective field (*H*_eff_ = 4*πNM*_s_‐2*K*_u_/*M*_s_) that highly relates to the demagnetization field energy. *H_K_*_1_ and *H_K_*_2_ are the anisotropy fields (*H_K_*_1_ = *K*_1_/*M*_s_, *H_K_*_2_ = *K*_2_/*M*_s_) that mainly depend on the magnetocrystalline anisotropy.^37^, ^38^, ^39^ *A*_1_(θ_M_), *A*_2_(θ_M_), *B*_1_(θ_M_), *B*_2_(θ_M_), *C*_1_(θ_M_), and *C*_2_(θ_M_) are decided by the film orientations, as shown in Table S1 in the Supporting Information. Figure 1h collects the *M*_s_ measured at room temperature by vibrating sample magnetometer systems. The corresponding M‐H loops are shown in Figure S3 in the Supporting Information. According to the formulas, the *H*_eff_, *H_K_*_1_, and *H_K_*_2_ can be obtained by fitting simultaneously the Kittel's resonance equations with the experimental values of *H*_r_ and ω/γ. In the inset of Figure 1h, the values of *H*_eff_, *H_K_*_1_, and *H_K_*_2_ are listed for the T‐LFO films, which are obtained by fitting the experimental data for the angular θ_H_‐dependent *H*_r_ with the formulas, as shown in Figure 1g. The formulas fit well with the experimental results of the T‐LFO (001), (110), and (111) films. It is clear that the contribution from *H*_eff_ dominates the MA properties of the T‐LFO films with a relatively high *M*_s_. When *M*_s_ is not large enough, the *F*_MAE_ influenced by the reduced symmetry of domain boundaries, interfaces, and roughness should have important effects on *H*_r_.^40^ More discussions on the contribution of *H_K_*_1_ and *H_K_*_2_ to the MA of the LFO films are given in the Supporting Information, which play the dominant role only at a relatively low *M*_s_. According to the previous reports, the substrate orientations usually play an important role in the formation of interfacial antiphase boundaries in spinel ferrite.^41^ Although all of the films were grown with almost the same thicknesses (≈150 nm), the different amount of interfacial defects could act as magnetic “dead” layers with different thicknesses, thus leading to the lowest *M*_s_ in T‐LFO (111) films.^42^ To further prove this, the T‐LFO (111)* film with poorer crystalline quality was fabricated by reducing the growth temperature from 650 to 550 °C, whose full width at half maximum (FWHM) of XRD rocking curve was boarder than that for T‐LFO (111), as shown in Figure S4 in the Supporting Information. (The growth condition was present in the Supporting Information.) The FMR spectra lines for the T‐LFO (111)* film were presented in Figure S5 in the Supporting Information. The nonzero noises at low scanning field come from the empty cavity, since FMR signals from the LFO (111)* samples are much weaker than the others. Although we failed to simulate the FMR line of LFO (111)* samples by the modified Dyson function due to the low SNR, the *H*_r_ can be roughly evaluated by using the zero passage of the FMR lines, as shown in Figure 1g. The corresponding fitting lines of *H*_r_, fitting parameters and *M*_s_ are also collected in Figure 1g,h. However, the fitting does not conduct very well, which called for the higher order of MA energy contributions for satisfied fitting. The T‐LFO (111)* film with poor crystalline quality showed lower MA in the FMR measurements, which further proved the main role that the *M*_s_ (or *H*_eff_) plays on the MA properties in the LFO films.

**Figure** **2**a displays the schematic illustration of FMR spectroscopy experimental set‐up for the sample under bending. The θ_H‐_dependent FMR spectra for the T‐LFO (001) film with unbent and bending states is shown in Figure 2b,c, respectively. As shown in the top left corner of Figure 2c, the bending axis is set along the y‐axis, which parallels to LFO [010] orientation in the whole bending film. The *x*‐axis and *z*‐axis are set along LFO [100] and [001] orientations of the central approximate nonbending unit cell, respectively. *R* represents the bending radius of the film, which is approximately the sum of the bending radius of the homemade bending mold and the thickness of the substrate. At a relatively small θ_H_ angle, the FMR spectra for the bent film retains the shape as the nearly uniform precession mode. When θ_H_ is higher than 40°, complex resonance modes gradually appeared, which are marked in Figure 2c. Figure 2d–g displays the contour plots of the θ_H_‐dependent integrated FMR absorption spectra for the T‐LFO (001) films with different bending states. Apparently increasing half width can be found in the bending films accompanied by the complex modes, which indicates the increasing loss. Moreover, it can be seen that the center of the red part in Figure 2d–g decreases with increasing of 1/*R*, which relates to the main resonance absorption peak of the film. After 1000 bending cycles (Figure 2h, in which the interruptions result from the low plot step of θ_H_ in the graph), the FMR spectrum retains its shape, showing excellent bending fatigue.

**Figure 2:**
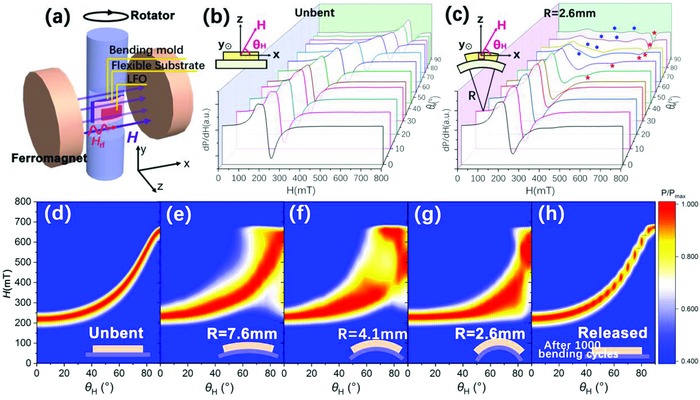
a) Schematic illustration of FMR spectroscopy experimental set‐up for the sample under bending. Angular θ_H_‐dependent FMR spectra for T‐LFO (001) film with b) unbent and c) bending status. d–h) Counter plot of the θ_H_‐dependent‐integrated FMR spectra for the T‐LFO (001) film with different bending states.

In order to explain the bending tuned FMR spectra of the LFO film, a simplified model is established. As shown in **Figure** **3**a, ∆φ represents the central angle of the bending film, and ∆*φ = C*/*R*, where *C* is the length of the film along the bending directions. For a specific sample, *C* is a constant. Then, *R* can be considered as ∆φ dependent only. If we divide a bending film into a great amount of parts defined by a small dφ, the film could be approximately regarded as the collection of various crystal domains with different orientations along the direction of applied ***H***. In order to facilitate calculation, here dφ is set as 1°. The overall dimensions of the samples and the corresponding calculation of ∆φ are present in the Table S3 in the Supporting Information. Thus, the final FMR spectra of the bent film could be considered as the sum of the FMR signals for the divided pieces of the thin film with different angles between the OOP orientation and the applied ***H***. In other word, the basis of this model is the misorientation effect induced by bending, which makes the plane of the divided pieces deviate from the initial position and changes the included angle between the plane of the divided pieces and the direction of applied ***H***. Following this consideration, we simulate the FMR spectra of the bending films through the mathematical integral operations on the data for unbent film using the closest ∆φ values, as shown in Figure 3b. Similar trend of graphic shape with decreasing *R*_s_ (*R*_s_ present the bending radius in the simulation, 1/*R*_s_ = ∆φ/*C*) has been found in the simulated bending data in Figure 3b, while the half width of the counter plot seems to be smaller than that for the experimental data in Figure 2e–g. Figure 3c displays the comparison of the experimental and the simulated result for a specific FMR spectra line obtained by the methods above. The simulated FMR lines show high similarity with the experimental FMR lines, especially for the position of the zero passages (*H_z_*). Although more than one *H_z_* value (*H_z_*_0_, *H_z_*_1_, *H_z_*_2_) could be found for the blue experimental FMR line in Figure 3c, there exists a set of very close *H_z_* values (*H_z_*_0_ in Figure 3c, which is defined as *H_z_*_c_) between the experimental and the simulated lines. Here, we argue that existence of *H_z_*_c_ should be attributed to the bending induced misorientation effect, which is also the main basis of the simulation. To further prove this, we list out all of *H_z_* values for the experimental FMR lines through a simple program, while that for the simulated lines is obtained by catching the maximum of the FMR absorption lines. Figure 3d–f collects the *H_z_* for the experimental and simulated FMR result. The *H_z_*_0_ (or *H_z_*_c_), *H_z_*_1_, *H_z_*_2_ in Figure 3c is marked in Figure 3d. For each θ_H_, a set of very close *H_z_* values (*H_z_*_c_) can always been found, which further proves the effect induced by the misorientation model. The *H_z_*_c_ for the experimental and simulated result is further listed in Figure 3g. It can be seen that the *H_z_*_c_ of the films shows an overall falling trend at relatively high θ_H_ values with increasing value of 1/*R*, which fits very well with the simulated curves. The simulated change of *H_z_*_c_ value (∆*H_z_*_c_) for T‐LFO (001) films under bending at θ_H_ ranging from 0° to 90° is present in Figure S6 in the Supporting Information, according to which it could be possible to predict the bending state of the film by comparing the *H_z_*_c_ data. The differences between the experimental and simulated *H_z_*_c_ values are collected in Figure S7d in the Supporting Information. However, in comparison with the simulated data, the experimental FMR spectra show higher loss as well as complex modes in details, as shown in Figure S7a–c in the Supporting Information, where the experimental and simulated FMR lines under different conditions are compared. Here, we note that the break of symmetry and the effective field fluctuations associated with the MA of randomly oriented domains in the bending film may be considered as two effective factors for the complex modes of the experimental results.^43^, ^44^ As shown in Figure 3a, the bending deformation induces a rotation of neighbor unit cell. From the volume perspective, every spin along the bending edge will deviate from its initial equilibrium orientation to accommodate spin flip over the spin chain, which might lead to the excitation of two‐magnon scattering (TMS) in the bending films.^43^ According to the previous reports, TMS is an important type of spin wave, and plays an important role in the magnetic relaxation mechanism for the limit of gigahertz excitations, which corresponds to the high loss in the bending films.^45^, ^46^ The tensile strain should also be one of the factors responsible for the complex modes in the bending films, which is reported to favor excitation of the surface spin‐wave mode.^47^

**Figure 3:**
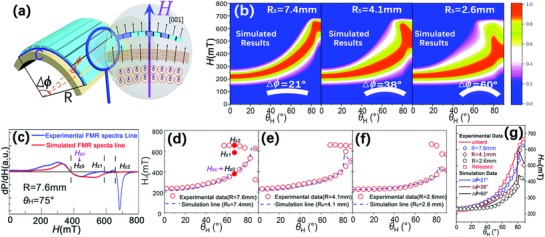
a) Diagram of the simple model for explaining the bending tuned FMR spectra of the LFO film. b) Counter plot of the simulated θ_H_‐dependent‐integrated FMR spectra for the T‐LFO (001) film with different bending status. Here, *R*_s_ = *C*/▵ϕ. c) Comparison of the experimental and simulated FMR spectra lines for T‐LFO films at θ_H_ = 75°, *R* = 7.6 mm. d–f) Experimental (scatter) and simulation curves (dash line) of angular θ_H_‐dependent *H*_z_ for T‐LFO (001) films under different bending states. g) Angular θ_H_‐dependent *H_z_*_c_ for T‐LFO (001) films under different bending states.

It is revealed that the mathematic simulation based on the misorientation effect can roughly predict the bending tuned *H_z_*_c_ of flexible LFO films. Because that the unbent T‐LFO (001) displays apparent MA in the FMR measurements (Figure 1g), the misorientation effect further induces the bending tenable *H_z_*_c_ during the tests. In other words, we argue that the bending tenability of *H_z_*_c_ should highly depend on the MA property (or *H*_eff_ values in Figure 1h) of the unbent LFO film. Figures S8 and Figure S9 in the Supporting Information show the experimental and simulated data of the bending tuned FMR spectra for T‐LFO (111), respectively, whose *H*_eff_ is lower than that for T‐LFO (001). Figure S10 in the Supporting Information displays the result for the T‐LFO (111)* with the lowest *H*_eff_ in the figure, whose SNR becomes too low to get precise simulated data. Compared with that for T‐LFO (001), the positions of the red parts of the integrated FMR spectra (the main FMR absorption peak) in Figure S8‐10 in the Supporting Information only change a little under bending, which proves the relationship between the MA property (or *H*_eff_ values) of the unbent LFO film and the bending tuned magnetic properties.

Besides the contribution from the crystalline orientation, for nanoscale materials, it is reported that the MA of materials can be further modulated by tuning the size and space distribution of the nano unit.^30^, ^48^ Hence, we consider fabricating LFO nanopillar arrays (NP‐LFO) on flexible F‐Mica substrates. As shown in **Figure** **4**a,b, the NP‐LFO produced by chemical etching method shows excellent morphology (see Experimental Section in the Supporting Information).^22^ The crystalline structure of NP‐LFO is ensured by the XRD results in Figure S11 in the Supporting Information, which also shows the (111) OOP orientation and IP multi‐domain structure. Figure 4c displays the M‐H loops and FMR spectra along both IP and OOP orientations for NP‐LFO, which shows excellent soft ferrimagnetism with the highest *M*_s_ among all LFO samples. The LFO film directly grown on the fluorophlogopite substrate (M‐LFO) with the same growth condition has also been measured for comparison. Modified Dyson function is applied to simulate the FMR spectra line and help find the *H*_r_. As shown in Figure 4d, a stable *H*_r_ is found in the NP‐LFO sample, whereas in the M‐LFO, there exists an apparent MA. The fitting curves calculated by Equations (2) and (3) fit very well to the experimental data. According to the fitting parameters collected in the inset table of Figure 4d, very low *H*_eff_ has been found in the NP‐LFO sample, which should be result from the low *N* (*H*_eff_ = 4*πNM*_s_ − *K*_u_/*M*_s_) in nanopillar structure.^30^ For nanoscale materials, *N* can be modulated by tuningthe size and space distribution of the nano unit.^30^, ^48^ Thus, according to the Equation (1), the decreasing of *N* (*N* < 1 for nanopillar/nanodot arrays, *N* = 1 for film) could effectively decrease the demagnetization field energy (4*πNM*_s_^2^sin^2^θ_M_), leading to the low maximum difference of *H*_r_ (MDR) in NP‐LFO samples. Figure 4e–h shows the counter plots of the θ_H_‐dependent‐integrated FMR spectra for the NP‐LFO (111) film with different bending states, which show nice stability under bending. It indicates that the low MA leads to the low tunability of *H_z_*_c_. Further works are required to investigate the relationship between the bending tuned FMR spectra and the structure‐related demagnetization factor *N*. Besides, the different bending deformation mechanism of the nanopillar arrays should also contribute to the stability under bending.^22^

**Figure 4:**
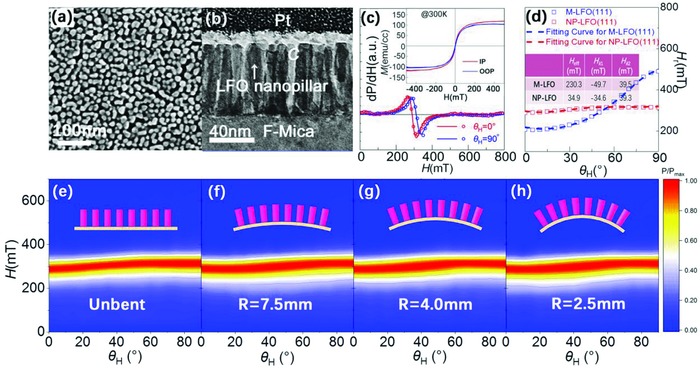
a) SEM image of the NP‐LFO (111) showing the surface morphology. b) The cross‐sectional TEM image showing the microstructure of the NP‐LFO (111). c) FMR spectra measured along IP and OOP orientations for the NP‐LFO (111). The scatter and the solid line are the experimental FMR data and the modified Dyson line, respectively. Inset of (c): corresponding M‐H loops of the NP‐LFO (111) measured at room temperature (300 K). d) Experimental (scatter) and fitting curves (dash line) of angular θ_H_‐dependent *H*_r_ for the NP‐LFO and the M‐LFO samples. Inset of (d) is a table, in which the fitting parameters *H*_eff_, *H_K_*_1_, and *H_K_*_2_ are listed. e–h) Counter plot of the θ_H_‐dependent‐integrated FMR spectra for the NP‐LFO (111) film with different bending states.

**Figure** **5**a concludes the relationship among the 4*πM*_s_, the fitted *H*_eff_ and MDR for all flexible LFO samples. It can be seen that the MDR increases with the increasing of *H*_eff_ for all samples, which is highly related to the demagnetization field energy. According to the former discussions, the high MDR relates to high bending tunability, and thus promises the application in bending sensors or flexible tunable resonators and filters, etc.^19^ A representative example could be the T‐LFO (001) film, as shown in the red line of Figure 5b. Bending could effective change the *H_z_*_c_ due to the disorientation effect in the bending T‐LFO film. The low MDR means relatively stable FMR spectra under bending, which could be further applied in flexible spintronics that demand stable properties. In fact, according to our results, the MDR of the discussed T‐LFO films is not low enough to meet the requirement of stable bending properties. Besides, low SNR induced by the poor ferrimagnetism accompanied with low *M*_s_ should also be avoided for future designs. Hence, NP‐LFO with low *H*_eff_ (or demagnetization field energy) is prepared, which not only displays the lowest MDR among all samples in this work, but also retains a high *M*_s_ to ensure the relatively high SNR. As shown in Figure 5b, the NP‐LFO (111) film nearly retains the values of *H_z_*_c_ under bending. From a practical point of view, nanopillar arrays is reported to have better bending flexible fatigue than films,^49^ which can be a good choice for flexible devices with stable properties under bending.

**Figure 5:**
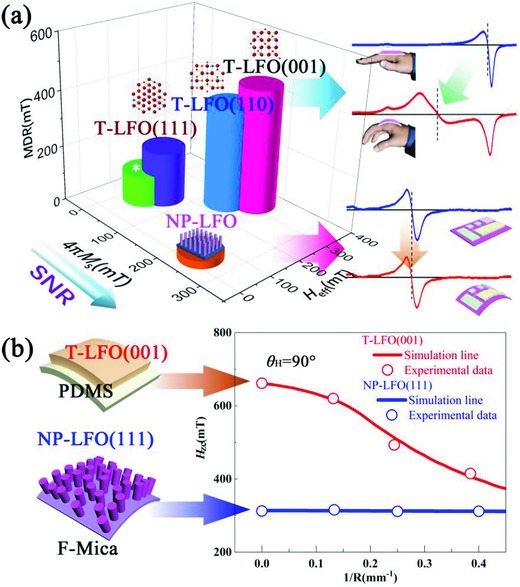
a) Diagram for the relationship among the 4*πM*_s_, *H*_eff_, MDR, and the promising application area of the LFO samples. b) The quantitative result of the bending radius *R*‐dependent *H*_zc_ for T‐LFO (001) and NP‐LFO (111) at θ_H_ = 90°.

In summary, the relationship between the bending tunability of *H_z_*_c_ and the MA properties of flexible LFO samples with different structures has been investigated and concluded on the basis of the FMR spectra tests. It is revealed that the MA properties of the T‐LFO films mainly contribute from the different *M*_s_ induced by the growth mechanism of LFO on substrates with different OOP orientations. The bending tunability of *H_z_*_c_ in the bending samples highly depends on the MA energy of the sample, and thus could be predicted by numerical simulations. With increasing MA energy, the *H_z_*_c_ of the LFO sample exhibits increasing tunability under bending, which shows the potential in the application of bending sensors or flexible tunable resonators and filters, etc. For the application of bending stable flexible devices that needs low MA energy, materials with high *M*_s_ as well as low *H*_eff_ (or demagnetization field energy) are required to ensure the high SNR. Thus, we obtain the NP‐LFO that holds the balance of low MA and high SNR. This work shows guiding significances for the design of future flexible tunable/stable microwave magnetic devices.

## Conflict of Interest

The authors declare no conflict of interest.

## Supporting information

Supplementary

## References

[1] H. E. Lee , S. Kim , J. Ko , H.‐I. Yeom , C.‐W. Byun , S. H. Lee , D. J. Joe , T.‐H. Im , S.‐H. K. Park , K. J. Lee , Adv. Funct. Mater. 2016, 26, 6170.

[2] M.‐C. Choi , Y. Kim , C.‐S. Ha , Prog. Polym. Sci. 2008, 33, 581.

[3] G. H. Gelinck , H. E. A. Huitema , E. van Veenendaal , E. Cantatore , L. Schrijnemakers , J. B. van der Putten , T. C. Geuns , M. Beenhakkers , J. B. Giesbers , B.‐H. Huisman , Nat. Mater. 2004, 3, 106. 14743215 10.1038/nmat1061

[4] H. Yu , C. C. Chung , N. Shewmon , S. Ho , J. H. Carpenter , R. Larrabee , T. L. Sun , J. L. Jones , H. Ade , B. T. O'Connor , F. So , Adv. Funct. Mater. 2017, 27, 1700461.

[5] Y. Yang , G. Yuan , Z. Yan , Y. Wang , X. Lu , J. M. Liu , Adv. Mater. 2017, 29, 1700425. 10.1002/adma.201700425 28449391

[6] S. R. Bakaul , C. R. Serrao , O. Lee , Z. Lu , A. Yadav , C. Carraro , R. Maboudian , R. Ramesh , S. Salahuddin , Adv. Mater. 2017, 29, 1605699. 10.1002/adma.201605699 28112840

[7] S. T. Han , H. Peng , Q. Sun , S. Venkatesh , K. S. Chung , S. C. Lau , Y. Zhou , V. A. L. Roy , Adv. Mater. 2017, 29, 1700375. 10.1002/adma.201700375 28671711

[8] W. W. Wu , B. Wang , M. Segev‐Bar , W. Dou , F. Niu , Y. D. Horev , Y. F. Deng , M. Plotkin , T. P. Huynh , R. Jeries , H. Zhu , A. Garaa , S. Badarneh , L. F. Chen , M. L. Du , W. W. Hu , H. Haick , Adv. Funct. Mater. 2017, 27, 1703147.

[9] M. C. McAlpine , H. Ahmad , D. Wang , J. R. Heath , Nat. Mater. 2007, 6, 379. 17450146 10.1038/nmat1891 PMC3695594

[10] Y. Zang , F. Zhang , C.‐a. Di , D. Zhu , Mater. Horiz. 2015, 2, 140.

[11] D. Wang , Y. Zhang , X. Lu , Z. Ma , C. Xie , Z. Zheng , Chem. Soc. Rev. 2018, 47, 4611. 29722373 10.1039/c7cs00192d

[12] D. Makarov , M. Melzer , D. Karnaushenko , O. G. Schmidt , Appl. Phys. Rev. 2016, 3, 011101.

[13] A. Bedoya‐Pinto , M. Donolato , M. Gobbi , L. E. Hueso , P. Vavassori , Appl. Phys. Lett. 2014, 104, 062412.

[14] H. Li , Q. Zhan , Y. Liu , L. Liu , H. Yang , Z. Zuo , T. Shang , B. Wang , R. W. Li , ACS Nano 2016, 10, 4403. 27032033 10.1021/acsnano.6b00034

[15] G. S. Canon Bermudez , D. D. Karnaushenko , D. Karnaushenko , A. Lebanov , L. Bischoff , M. Kaltenbrunner , J. Fassbender , O. G. Schmidt , D. Makarov , Sci. Adv. 2018, 4, eaao2623. 29376121 10.1126/sciadv.aao2623 PMC5777399

[16] M. Gueye , B. M. Wague , F. Zighem , M. Belmeguenai , M. S. Gabor , T. Petrisor , C. Tiusan , S. Mercone , D. Faurie , Appl. Phys. Lett. 2014, 105, 062409.

[17] Y. Zhang , L. Shen , M. Liu , X. Li , X. Lu , L. Lu , C. Ma , C. You , A. Chen , C. Huang , L. Chen , M. Alexe , C. L. Jia , ACS Nano 2017, 11, 8002. 28657728 10.1021/acsnano.7b02637

[18] H. J. Liu , C. K. Wang , D. Su , T. Amrillah , Y. H. Hsieh , K. H. Wu , Y. C. Chen , J. Y. Juang , L. M. Eng , S. U. Jen , Y. H. Chu , ACS Appl. Mater. Interfaces 2017, 9, 7297. 28155267 10.1021/acsami.6b16485

[19] W. L. Liu , M. Liu , R. Ma , R. Y. Zhang , W. Q. Zhang , D. P. Yu , Q. Wang , J. N. Wang , H. Wang , Adv. Funct. Mater. 2018, 28, 1705928.

[20] L. Shen , L. Wu , Q. Sheng , C. Ma , Y. Zhang , L. Lu , J. Ma , J. Ma , J. Bian , Y. Yang , A. Chen , X. Lu , M. Liu , H. Wang , C. L. Jia , Adv. Mater. 2017, 29, 1702411. 10.1002/adma.201702411 28639318

[21] P. C. Wu , P. F. Chen , T. H. Do , Y. H. Hsieh , C. H. Ma , T. D. Ha , K. H. Wu , Y. J. Wang , H. B. Li , Y. C. Chen , J. Y. Juang , P. Yu , L. M. Eng , C. F. Chang , P. W. Chiu , L. H. Tjeng , Y. H. Chu , ACS Appl. Mater. Interfaces 2016, 8, 33794. 27960370 10.1021/acsami.6b11610

[22] L. Shen , M. Liu , C. Ma , L. Lu , H. Fu , C. You , X. Lu , C.‐L. Jia , Mater. Horiz. 2018, 5, 230.

[23] Q. Gan , R. A. Rao , C. B. Eom , J. L. Garrett , M. Lee , Appl. Phys. Lett. 1998, 72, 978.

[24] Y. Bitla , Y.‐H. Chu , FlatChem 2017, 3, 26.

[25] C. Zhou , L. K. Shen , M. Liu , C. X. Gao , C. L. Jia , C. J. Jiang , Phys. Rev. Appl. 2018, 9, 014006.

[26] M. Golosovsky , P. Monod , P. K. Muduli , R. C. Budhani , Phys. Rev. B 2007, 76, 184413.

[27] A. V. Chumak , V. I. Vasyuchka , A. A. Serga , B. Hillebrands , Nat. Phys. 2015, 11, 453.

[28] N. Pachauri , B. Khodadadi , M. Althammer , A. V. Singh , B. Loukya , R. Datta , M. Iliev , L. Bezmaternykh , I. Gudim , T. Mewes , A. Gupta , J. Appl. Phys. 2015, 117, 233907.

[29] R. Zhang , M. Liu , L. Lu , S.‐B. Mi , H. Wang , CrystEngComm 2015, 17, 8256.

[30] S. Noh , D. Monma , K. Miyake , M. Doi , T. Kaneko , H. Imamura , M. Sahashi , IEEE Trans. Magn. 2011, 47, 2387.

[31] U. Nowosielecka , R. Pelka , I. Moszyńska , N. Guskos , J. Typek , G. Żołnierkiewicz , J. Magn. Magn. Mater. 2017, 443, 324.

[32] V. Popovych , M. Bester , I. Stefaniuk , M. Kuzma , Nukleonika 2015, 60, 385.

[33] J. P. Joshi , S. V. Bhat , J. Magn. Reson. 2004, 168, 284. 15140439 10.1016/j.jmr.2004.03.018

[34] S. Lee , S. Grudichak , J. Sklenar , C. C. Tsai , M. Jang , Q. Yang , H. Zhang , J. B. Ketterson , J. Appl. Phys. 2016, 120, 033905.

[35] M. Gueye , F. Zighem , M. Belmeguenai , M. Gabor , C. Tiusan , D. Faurie , J. Phys. D: Appl. Phys. 2016, 49, 265001.

[36] J. Smit , H. G. Beljers , Philips Res. Rep. 1955, 10, 113.

[37] V. Dyakonov , V. Shapovalov , E. Zubov , P. Aleshkevych , A. Klimov , V. Varyukhin , V. Pashchenko , V. Kamenev , V. Mikhailov , K. Dyakonov , V. Popov , S. J. Lewandowski , M. Berkowski , R. Zuberek , A. Szewczyk , H. Szymczak , J. Appl. Phys. 2003, 93, 2100.

[38] O. Kohmoto , J. Magn. Magn. Mater. 2003, 262, 280.

[39] O. Kohmoto , Jpn. J. Appl. Phys. 2003, 42, 7299.

[40] M. Farle , Rep. Prog. Phys. 1998, 61, 755.

[41] C. Gatel , B. Warot‐Fonrose , S. Matzen , J. B. Moussy , Appl. Phys. Lett. 2013, 103, 092405.

[42] E. J. Guo , T. Charlton , H. Ambaye , R. D. Desautels , H. N. Lee , M. R. Fitzsimmons , ACS Appl. Mater. Interfaces 2017, 9, 19307. 28509529 10.1021/acsami.7b03252

[43] X. Xue , Z. Zhou , G. Dong , M. Feng , Y. Zhang , S. Zhao , Z. Hu , W. Ren , Z. G. Ye , Y. Liu , M. Liu , ACS Nano 2017, 11, 9286. 28813600 10.1021/acsnano.7b04653

[44] M. Collet , X. de Milly , O. d. A. Kelly , V. V. Naletov , R. Bernard , P. Bortolotti , J. Ben Youssef , V. E. Demidov , S. O. Demokritov , J. L. Prieto , M. Munoz , V. Cros , A. Anane , G. de Loubens , O. Klein , Nat. Commun. 2016, 7, 10377. 26815737 10.1038/ncomms10377 PMC4737803

[45] A. Azevedo , A. B. Oliveira , F. M. de Aguiar , S. M. Rezende , Phys. Rev. B 2000, 62, 5331.

[46] K. Zakeri , J. Lindner , I. Barsukov , R. Meckenstock , M. Farle , U. von Hoersten , H. Wende , W. Keune , J. Rocker , S. S. Kalarickal , K. Lenz , W. Kuch , K. Baberschke , Z. Frait , Phys. Rev. B 2007, 76, 104416.

[47] V. Shapovalov , V. Dyakonov , P. Aleshkevych , K. Dyakonov , I. Zhikharev , M. Kuzminski , H. Szymczak , Phys. Status Solidi B 2007, 244, 347.

[48] J. Salado , M. Insausti , L. Lezama , I. G. de Muro , E. Goikolea , T. Rojo , Chem. Mater. 2011, 23, 2879.

[49] S. Merabtine , F. Zighem , D. Faurie , A. Garcia‐Sanchez , P. Lupo , A. O. Adeyeye , Nano Lett. 2018, 18, 3199. 29668289 10.1021/acs.nanolett.8b00922

