# Supplementary material for: A Strategy to Modulate the Bending Coupled Microwave Magnetism in Nanoscale Epitaxial Lithium Ferrite for Flexible Spintronic Devices
Source: Adv Sci (Weinh). 2018 Nov 6;5(12):1800855. doi: 10.1002/advs.201800855 (PMC6299733; doi:10.1002/advs.201800855)
Supplement: Supplementary file 1 — Supplementary [file ADVS-5-1800855-s001.pdf]

Copyright WILEY-VCH Verlag GmbH & Co. KGaA, 69469 Weinheim, Germany, 2018.

ADVANCED SCIENCE
Open Access

Supporting Information

for *Adv. Sci.,* DOI: 10.1002/advs.201800855

A Strategy to Modulate the Bending Coupled Microwave Magnetism in Nanoscale Epitaxial Lithium Ferrite for Flexible Spintronic Devices

Lvkang Shen, Guohua Lan, Lu Lu, Chunrui Ma, Cuimei Cao, Changjun Jiang, Huarui Fu, Caiyin You, Xiaoli Lu, Yaodong Yang, Lang Chen, Ming Liu, and Chun-Lin Jia*

Copyright WILEY-VCH Verlag GmbH & Co. KGaA, 69469 Weinheim, Germany, 2018.

Supporting Information

for *Adv. Sci.*, DOI: 10.1002/advs.201800855

A Strategy to Modulate the Bending Coupled Microwave Magnetism in Nanoscale Epitaxial Lithium Ferrite for Flexible Spintronic Devices

Lvkang Shen, Guohua Lan, Lu Lu, Chunrui Ma, Cuimei Cao, Changjun Jiang, Huarui Fu, Caiyin You, Xiaoli Lu, Yaodong Yang, Lang Chen, Ming Liu, Chun-Lin Jia*

Experimental Section

***Fabrication of the Epitaxial Lithium Ferrite Films*:** The $LiFe_5O_8$ (LFO) films (~150nm) with different out-of-plane (OOP) crystal orientations were grown on 40nm $La_{0.67}Sr_{0.33}MnO_3$ (LSMO) buffered $SrTiO_3$ (STO) substrates. A KrF excimer pulsed laser deposition system with a wavelength of 248 nm was employed to fabricate the epitaxial LFO/LSMO double layer films. The targets of LSMO and LFO were prepared through a standard ceramic sintering process.[1, 2] The energy density of the laser beam focused onto the target was about 2.0 J/cm^2. Then, the LSMO film layers were grown at 700˚C with an oxygen pressure of 250 mTorr. The LFO thin films (LFO (001), LFO (110) and LFO (111)*) were directly deposited onto the LSMO layers at 550 ˚C with an oxygen pressure of 50mTorr. The LFO (111) films were grown at 650 ˚C with an oxygen pressure of 50 mTorr. After the growth of LFO/LSMO double layer films, they were annealed at 550 ˚C for 15 min under an oxygen of 400 Torr, and then slowly cooled down to room temperature at the rate of 5 ˚C /min.

***Film transfer method*:** Approximately 0.1-mm-thick polydimethylsiloxane (PDMS, Dowcorning 184SYLGAR) was spread on a clean glass wafer to make flat PDMS sheets. After the film growth process, the LFO/LSMO/STO structure was firstly attached onto the PDMS sheets and then put into the KI+HCl solutions to etch out the LSMO layer.[3] After 24

hours, the LSMO layer got etched out, and the LFO layer was peeled off from the STO substrate. Hence, we got the LFO/PDMS samples, which are shown in Figure S1.

***Fabrication of the Lithium Ferrite Nanopillar arrays*:** The LFO nanopillar arrays (NP-LFO) were fabricated through etching out the MgO phase of the LFO:MgO nanocomposite thin films on fluorophlogopite mica (F-Mica) substrates. The target of LFO:MgO for film deposition was prepared by mixing up the high purity LFO and MgO powders with a volume ratio of 1:2 and then sintered at 1000°C. The epitaxial LFO:MgO nanocomposite thin films were fabricated on F-Mica substrate by employing a KrF excimer pulsed laser deposition system with the wavelength of 248 nm. The energy density of the laser beam focused onto the target was about 2.0 J/cm^2. The nanocomposite film was grown at 800 ˚C with an oxygen pressure of 20 mTorr. After laser deposition, it was annealed at 800 ˚C for 15 min under an oxygen pressure of 400 Torr, and then slowly cooled down to room temperature. In order to etch out the MgO phases, the nanocomposite films are put into a 10 % $(NH_4)_2SO_4$ solution in a water-bath at 60 °C for ~6 hours, leaving only (111)-oriented epitaxial LFO nanopillar arrays standing on the flexible F-Mica substrates.

***Characterizations*:** The crystallinity of the as-grown (A-LFO), transferred films (T-LFO) and LFO nanopillar arrays (NP-LFO) was characterized by HRXRD on PANalytical X'Pert MRD. The microstructure and morphology of the LFO nanopillar (NP-LFO) were investigated by transmission electron microscope (TEM JEM-2100) and scanning electron microscope (SEM Quanta F250). The NP-LFO arrays were firstly filled by carbon and then coated by Pt for better resolution in TEM test. The magnetic hystersis loops were measured at room temperature (300K) by the vibrating sample magnetometer of Lake Shore. The electron spin resonance (ESR) measurements were performed using the JEOL, JES-FA 300 spectrometer (X-band at 9.0 GHz, power of 1 mW). The ferromagnetic resonance (FMR) absorption spectra were measured using a lock-in technique based on sweeping of a static external magnetic field superimposed over the ac magnetic field. Measurements for the bending

samples were carried out by attaching the samples to acrylonitrile butadiene styrene (ABS) plastic rods with different radius from 2.5 mm to 7.5 mm. The rods were cut as molds with less than 1 mm thick to ensure that all samples were put in a uniform field during the FMR test.

***Bending Cycling*:** One side of the PDMS substrate is fixed on the plastic rod (radius=4 mm), while the other side is attached and then peeled as a bending cycling. Hence, we repeat this process for 1000 times.

Derivation of the F_{MAE}, the F_{ME} and the Fitting Equations

***The Magnetocrystalline Anisotropy Energies F_{MAE}*:** According to the previous reports,[4] the magnetocrystalline anisotropy energies F_{MAE} can be expressed by the anisotropy constant K_1 and K_2:

$$F_{\mathrm{MAE}}=K_1(\alpha_u^2\alpha_v^2+\alpha_u^2\alpha_w^2+\alpha_v^2\alpha_w^2)+K_2(\alpha_u^2\alpha_v^2\alpha_w^2) \quad .(1)$$

Here α_u, α_v and α_w are the vector components of the magnetization ***M*** along the basic axes of crystal coordinates: *u*[001], *v*[010], and *w*[001]. Considering the transformation between the crystal coordinates (*u*, *v*, *w*) and the coordinates defined for the film systems (x, y, z shown in the bottom of Figure 1d-f and the right corner of Figure 1g), α_u, α_v and α_w can be expressed as:

$$\alpha_u=cos\theta_{\mathrm{M}}cos\varphi_{\mathrm{M}}cos\alpha_1+cos\theta_{\mathrm{M}}sin\varphi_{\mathrm{M}}cos\beta_1+sin\theta_{\mathrm{M}}cos\gamma_1 \quad ;(2)$$

$$\alpha_v=cos\theta_{\mathrm{M}}cos\varphi_{\mathrm{M}}cos\alpha_2+cos\theta_{\mathrm{M}}sin\varphi_{\mathrm{M}}cos\beta_2+sin\theta_{\mathrm{M}}cos\gamma_2 \quad ;(3)$$

$$\alpha_w=cos\theta_{\mathrm{M}}cos\varphi_{\mathrm{M}}cos\alpha_3+cos\theta_{\mathrm{M}}sin\varphi_{\mathrm{M}}cos\beta_3+sin\theta_{\mathrm{M}}cos\gamma_3 \quad .(4)$$

$\alpha_1/\beta_1/\gamma_1$ are the included angles between x and *u*/*v*/*w*. $\alpha_2/\beta_2/\gamma_2$ are the included angles between y and *u*/*v*/*w*. $\alpha_3/\beta_3/\gamma_3$ are the included angles between z and *u*/*v*/*w*. Thus, F_{MAE} represents the anisotropy energies for the epitaxial films with different in-plane (IP) and out-of-plane (OOP) crystallographic orientations. Table S2 shows the expressions of α_u, α_v and α_w with the crystal orientation.

***The Fitting Equations*:** The expression for the FMR frequency can be derived by

$$\left(\frac{\omega}{\gamma}\right)^2=\frac{1}{M_{\mathrm{s}}^2cos^2\theta_{\mathrm{M}}}\left[\frac{\partial^2F}{\partial\theta_{\mathrm{M}}^2}\frac{\partial^2F}{\partial\varphi_{\mathrm{M}}^2}-\left(\frac{\partial^2F}{\partial\theta_{\mathrm{M}}\partial\varphi_{\mathrm{M}}}\right)^2\right] \quad ;(5)$$

$$\frac{1}{M_{\mathrm{s}}}\frac{\partial F}{\partial\theta_{\mathrm{M}}}=0 \quad ;(6)$$

$$\frac{1}{M_{\mathrm{s}}}\frac{\partial F}{\partial\varphi_{\mathrm{M}}}=0 \quad .(7)$$

Finally, we obtained the expressions in the main text as the Equation (2) and (3) in the manuscript, which were used to fit the experimental results based on the parameters H_{eff}, H_{K1} and H_{K2}.

What should be mentioned is that for the LFO samples with (111) OOP orientations (T-LFO (111), T-LFO (111)*, M-LFO (111) and NP-LFO (111)), multiple IP orientations have been found in the LFO domains by the XRD φ-scan. Considering the symmetry of the crystal structure, here we fit the experimental data approximately by using the method for single crystalline LFO samples with IP (11$\bar{2}$) orientation.

***The Magnetoelastic Energies F_{ME}*:** To simplify the result, here we only discussed the magnetoelastic energy for the LFO with (001) OOP orientation. The magnetoelastic energy was deduced using the orthogonal normal stress model by M. Gueyue et al.[5]:

$$F_{\mathrm{ME}}=-\frac{3}{2}\lambda(\sigma_{\mathrm{xx}}cos^2\theta_{\mathrm{M}}cos^2\varphi_{\mathrm{M}}+\sigma_{\mathrm{yy}}cos^2\theta_{\mathrm{M}}sin^2\varphi_{\mathrm{M}}+\sigma_{\mathrm{zz}}sin^2\theta_{\mathrm{M}})+\frac{\lambda}{2}(\sigma_{\mathrm{xx}}+\sigma_{\mathrm{yy}}+\sigma_{\mathrm{zz}}) \quad (8)$$

where σ_{xx}, σ_{yy} and σ_{zz} are the orthogonal normal stresses along x, y and z axis, respectively. λ is the isotropic magnetostriction coefficient (λ_{100}= -27.8ppm).

In this paper, the 150-nm-thick transferred LFO film on the PDMS substrate should be relaxed according to the XRD result and the former reports.[3] Therefore, the magnetoelastic energy should not contribute a lot to the FMR result of the unbent film. Here, we mainly discuss the pure strain induced by bending, in order to figure out whether it can influence the MA in FMR measurements or not. Normally, the strain induced by bending can be calculated as:

$$\varepsilon_{\mathrm{yy}} = \varepsilon_{\mathrm{eff}}=\left(\frac{t}{2r}\right)\times\frac{(1+2\eta+\chi\eta^2)}{(1+\eta)(1+\chi\eta)}=\left(\frac{t}{2r}\right)\times\left(1+\frac{(1-\chi)\eta}{(1+\eta)(1+\chi\eta)}\right) \quad (9)$$

Where t is the total thicknesses of the LFO film and the PDMS tape (t=150nm+100 μm≈100 μm), η is the thickness ratio of the LFO film to the PI substrate (η=0.0015), χ is elastic moduli ratio between the LFO film and the PI substrate ($\chi \approx 17400$). Then we can obtain the relationship between the bending radius r and the strain along the the bending axis y ($\varepsilon_{\mathrm{yy}}$). In the bending model, $\varepsilon_{\mathrm{xx}}=\varepsilon_{\mathrm{zz}}=-v\varepsilon_{\mathrm{yy}}$, where $v\approx0.3$. $\varepsilon_{\mathrm{xx}}$ and $\varepsilon_{\mathrm{zz}}$ present the strain along x and z axis, respectively. The Young's modulus Y along the [100] direction of the LFO is 1.74×10^{12} dyne cm^{-2}. Hence, the F_{ME} can be characterized as a function of r, θ_{H}, φ_{M}:

$$F_{\mathrm{ME}}=[\frac{3\lambda}{2}(v cos^2\theta_{\mathrm{M}} cos^2\varphi_{\mathrm{M}}\text{-}cos^2\theta_{\mathrm{M}} sin^2\varphi_{\mathrm{M}}+v sin^2\theta_{\mathrm{M}})+\frac{\lambda}{2}(1\text{-}2v)]\left(\frac{t}{2}\right)\left(1+\frac{(1\text{-}\chi)\eta}{(1+\eta)(1+\chi\eta)}\right)\left(\frac{1}{r}\right) \quad (10)$$

By plugging the parameters for LFO (001) that we discussed above in Equation (11) and (12) in the Supporting Information, the equation can be further derived as:

$$\left(\frac{\omega}{\gamma}\right)^2=\left[H cos(\theta_{\mathrm{M}}\text{-}\theta_{\mathrm{H}})+4\pi M_{\mathrm{s}} cos(2\theta_{\mathrm{M}})\text{-}\frac{2K_{\perp}}{M_{\mathrm{s}}}cos2\theta_{\mathrm{M}}+\frac{2K_1}{M_s}cos4\theta_{\mathrm{M}}\right]\times$$
$$\left[H cos(\theta_{\mathrm{M}}\text{-}\theta_{\mathrm{H}})\text{-}4\pi M_{\mathrm{s}} sin^2\theta_{\mathrm{M}}+\frac{2K_{\perp}}{M_{\mathrm{s}}}sin^2\theta_{\mathrm{M}}+\frac{K_1}{2M_{\mathrm{s}}}(3+cos4\theta_{\mathrm{M}})+\frac{K_2}{2M_{\mathrm{s}}}sin^2(2\theta_{\mathrm{M}})+\frac{3\lambda Y}{M_{\mathrm{s}}}(2v\text{-}1)\frac{(1+2\eta+\chi\eta^2)}{(1+\eta)(1+\chi\eta)}\left(\frac{t}{2r}\right)\right] \quad (11)$$

$$\frac{1}{M_{\mathrm{s}}}\frac{\partial F}{\partial\theta_{\mathrm{M}}}=H\sin(\theta_{\mathrm{M}}\text{-}\theta_{\mathrm{H}})+2\pi M_{\mathrm{s}}\sin2\theta_{\mathrm{M}}\text{-}\frac{K_{\perp}}{M_{\mathrm{s}}}sin2\theta_{\mathrm{M}}+\frac{K_1}{2M_{\mathrm{s}}}sin4\theta_{\mathrm{M}}=0 \quad (12)$$

Then, based on the formulas, the simulation of the angular θ_{M} dependent H_{r} for the T-LFO (001) film under different degrees of pure tensile strain along *y* axis was present in Figure S12. It reveals that the pure tensile strain along *y* axis can theoretically decrease the H_{r} of the LFO film, which is similar to the result by W. Liu et al. However, the pure tensile strain might not have a huge influence on the property of the bending film, due to the poor transfer efficiency of strain. [6] Even for the film under a bending radius of ~2.6 mm, the effective strain $\varepsilon_{\mathrm{eff}}$ is calculated to be less than 0.03%, and the corresponding change is much smaller than that calculated from bending induced misorientation effects. Therefore, we argue that the F_{ME} should not be the main cause for the bending modulated H_{r} in our experiment.

Discussion about the contribution from H_{k1} and H_{k2} to the MA of LFO films

H_{K1} and H_{K2} are the anisotropy fields that mainly depend on the magnetocrystalline anisotropy. Firstly, according to the Equations (2) and (3) in the manuscript, it can influence the MA of LFO films. As shown in Figure S13a, where we provide the simulation result for T-LFO (001) with and without considering the contribution from H_{K1} and $H_{K2.}$

Secondly, according to Table S1, the different crystalline orientation could influence the MA properties in FMR, even with the same value of H_{K1} and H_{K2}. Figure S13b presents the simulation results for T-LFO (001) and T-LFO (110) using the same parameters from Figure 1h, which are different. The differences become larger when H_{eff} becomes smaller, as shown in Figure S13c. Ideally, K_1 and K_2 should be constant for the specific materials. Therefore, H_{K1} and H_{K2} increase with decreasing M_s. It reveals that the crystal orientation play an important role on the MA properties only when M_s is low so that H_{k1} and H_{k2} could be large enough.

In this paper, although films with the same thicknesses and different orientations are provided, the differences in M_s are very large due to the different growth mechanism. Therefore, we argue that it is not a feasible method to modulate the MA in FMR by just changing the crystalline orientation of the film.

References

[1] C. Yang, F. Wang, C. Zhang, C. Zhou, C. Jiang, *J. Phys. D: Appl. Phys.* **2015**, 48, 435001.

[2] S. Majumdar, H. Huhtinen, H. Majumdar, R. Laiho, R. Österbacka, *J. Appl. Phys.* **2008**, 104, 033910.

[3] L. Shen, L. Wu, Q. Sheng, C. Ma, Y. Zhang, L. Lu, J. Ma, J. Ma, J. Bian, Y. Yang, A. Chen, X. Lu, M. Liu, H. Wang, C. L. Jia, *Adv. Mater.* **2017**, 29, 1702411.

[4] C. Chappert, K. L. Dang, P. Beauvillain, H. Hurdequint, D. Renard, *Phys. Rev. B* **1986**, 34, 3192.

[5] M. Gueye, F. Zighem, M. Belmeguenai, M. Gabor, C. Tiusan, D. Faurie, *J. Phys. D: Appl. Phys.* **2016**, 49, 265001.

[6] W. L. Liu, M. Liu, R. Ma, R. Y. Zhang, W. Q. Zhang, D. P. Yu, Q. Wang, J. N. Wang, H. Wang, *Adv. Funct. Mater.* **2018**, 28, 1705928.

Table S1 $A_1(\theta_M)$, $A_2(\theta_M)$, $B_1(\theta_M)$, $B_2(\theta_M)$, $C_1(\theta_M)$, $C_2(\theta_M)$ for different OOP orientations

OOP crystal orientation		$A_1(\theta_M)$	$A_2(\theta_M)$
(001)	IP(100)	$2cos4\theta_M$	0
(110)	IP(001)	$(cos2\theta_M+3cos4\theta_M)/2$	$(cos2\theta_M+8cos4\theta_M-9cos6\theta_M)/32$
(111)	IP(11-2)	$(cos2\theta_M-7cos4\theta_M-2\sqrt{2}sin2\theta_M -4\sqrt{2}sin4\theta_M)/6$	$-(51cos2\theta_M+ 24\sqrt{2}sin(2\theta_M) + 8\sqrt{2}sin(4\theta_M) - 30\sqrt{2}sin^3 2\theta_M+ 28cos^2 2\theta_M - 69cos^3 2\theta_M - 14)/72$

OOP crystal orientation		$B_1(\theta_M)$	$B_2(\theta_M)$
(001)	IP(100)	$(3+cos4\theta_M)/2$	$(sin^2 2\theta_M)/2$
(110)	IP(001)	$(16cos2\theta_M+3cos4\theta_M-3)/8$	$(14cos4\theta_M+3cos2\theta_M-3cos6\theta_M -14)/64$
(111)	IP(11-2)	$16cos2\theta_M-7cos4\theta_M -32\sqrt{2}sin2\theta_M -4\sqrt{2}sin4\theta_M-9)/24$	$-(1/18)\, cos\theta_M\, (- 6\sqrt{2}sin\theta_M + \sqrt{2}\, cos^5\theta_M tan\theta_M+cos^4\theta_M sin\theta_M tan\theta_M + 2\sqrt{2}\, sin^5\theta_M +8\, sin^5\theta_M tan\theta_M + cos^3\theta_M\, (6 -7\sqrt{2}\, sin^2\theta_M\, tan\theta_M) + cos^2\theta_M\, (15\sqrt{2}\, sin\theta_M - 14\, sin^3\theta_M\, tan\theta_M))$

OOP crystal orientation		$C_1(\theta_M)$	$C_2(\theta_M)$
(001)	IP(100)	$sin4\theta_M/2$	0
(110)	IP(001)	$(2sin2\theta_M+3sin4\theta_M)/8$	$(sin2\theta_M+4sin4\theta_M-3sin6\theta_M)/64$
(111)	IP(11-2)	$(2sin2\theta_M-7sin4\theta_M +4\sqrt{2}cos2\theta_M +4\sqrt{2}cos4\theta_M)/24$	$cos\theta_M(\sqrt{2}cos\theta_M - 4sin\theta_M)(\sqrt{2}cos\theta_M- sin\theta_M)(\sqrt{2}cos\theta_M+2sin\theta_M)^3/72$

Table S2 α_u, α_v, α_w values for the crystal orientation discussed in the manuscript.

OOP crystal orientation		α_u	α_v	α_w
(001)	IP(100)	$cos\theta_M cos\varphi_M$	$cos\theta_M sin\varphi_M$	$sin\theta_M$
(110)	IP(001)	$sin\theta_M$	$(cos\theta_M cos\varphi_M - cos\theta_M sin\varphi_M)/\sqrt{2}$	$(cos\theta_M cos\varphi_M + cos\theta_M sin\varphi_M)/\sqrt{2}$
(111)	IP(11-2)	$cos\theta_M cos\varphi_M/\sqrt{6} - cos\theta_M sin\varphi_M/\sqrt{2} + sin\theta_M/\sqrt{3}$	$cos\theta_M cos\varphi_M/\sqrt{6} + cos\theta_M sin\varphi_M/\sqrt{2} + sin\theta_M/\sqrt{3}$	$-2cos\theta_M sin\varphi_M/\sqrt{6} + sin\theta_M/\sqrt{3}$

Table S3 overall dimensions of the LFO samples and the calculated R_s and Δ□ discussed in this work.

Samples	x(mm)	y(mm)[*1]	z(mm)[*2]	Δ□(°) / R_s(mm)[*3]		
				R_1=7.5mm	R_2=4.0mm	R_3=2.5mm
T-LFO (001)	2.50	2.72	0.10	21 / 7.42	38 / 4.10	60 / 2.59
T-LFO (111)	2.43	2.73	0.10	21 / 7.45	38 /4.12	60 / 2.61
NP-LFO (111)	2.48	2.55	0.02	N/A[*4]	N/A	N/A

[*1] y is the C in the manuscript, which represents the length of the film along the bending directions.

[*2] z is the thickness of the total nanostructure and substrate system, which approximately equals to the thickness of the substrate

[*3] Here $\Delta\phi \approx y/R$ ($R=R_1+z$, R_2+z or R_3+z. R_1, R_2 and R_3 are the bending radii of the different homemade bending molds). The step of $\Delta\phi$ is limited to 1° in this work. Therefore, we can't always obtain the Rs value that equals to R

[*4] The simulation for NP-LFO (111) is not provided in this paper. Therefore, this part is neglected here.

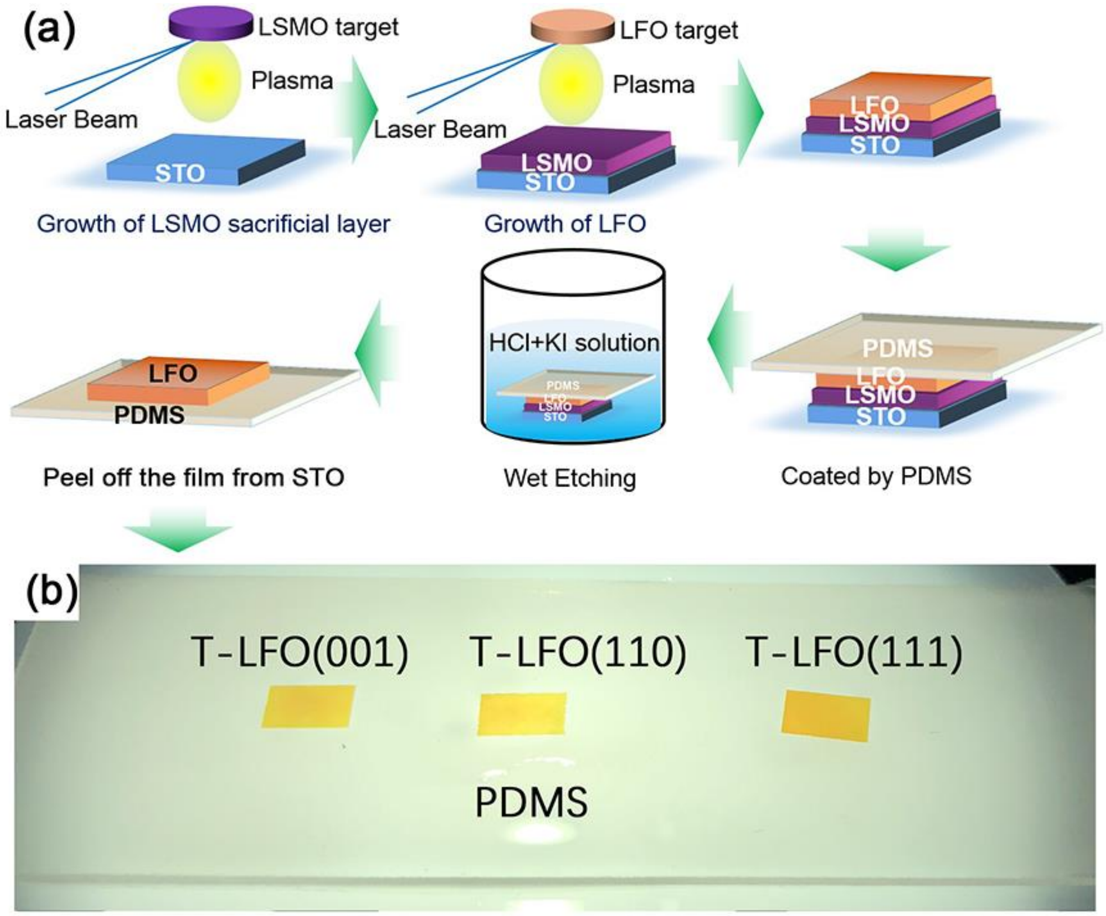

Figure S1. (a) The synthesis process of transferred lithium ferrite (T-LFO) thin films on flexible PDMS substrates. (b) Optical photographs of transferred lithium ferrite (T-LFO) thin films with different out-of-plane orientations.

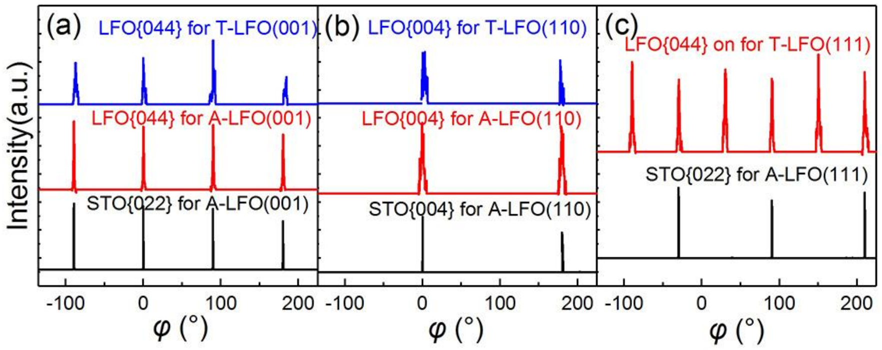

Figure S2. (a-c**)** Typical XRD φ-scan results for A-LFO and T-LFO with (a) (001), (b) (110) and (c) (111) OOP orientations, respectively.

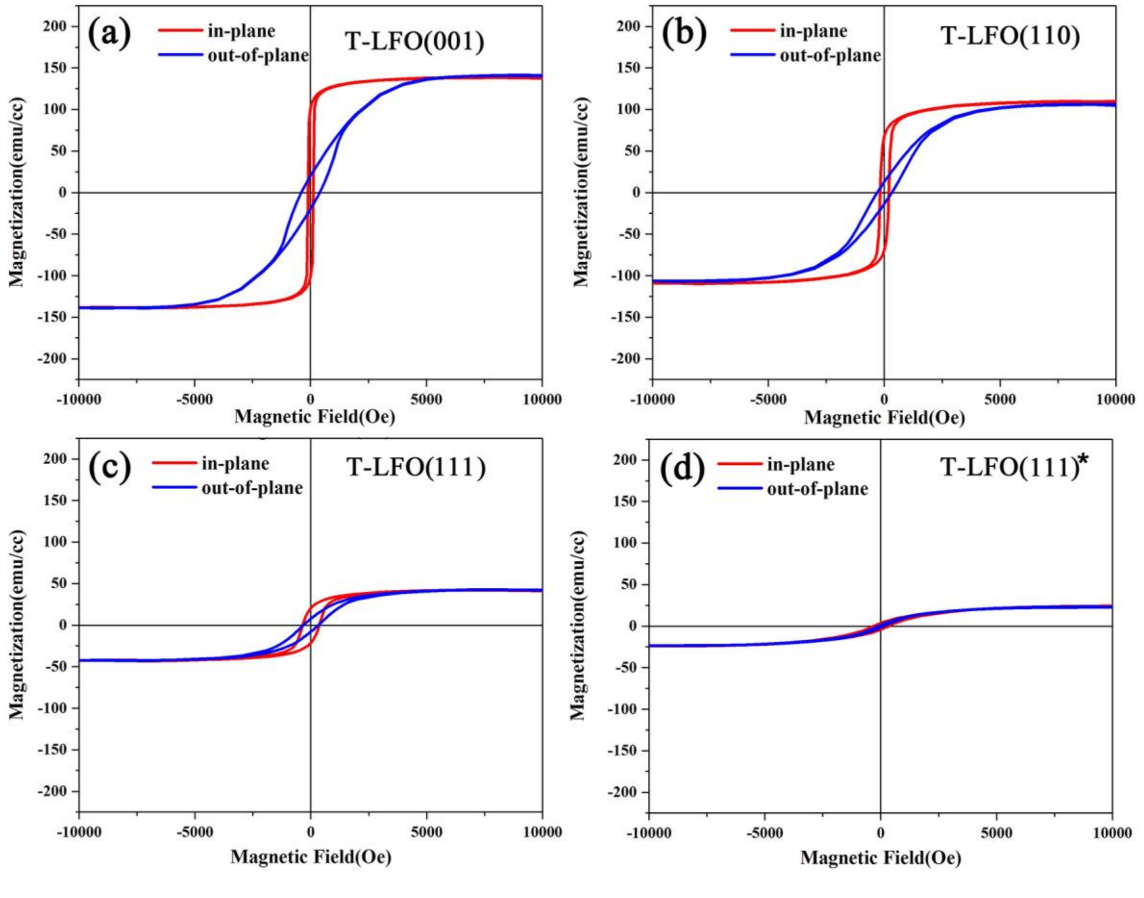

Figure S3. (a-d) *M-H* loops measured at room temperature (300K) along OOP and IP directions of (a) T-LFO (001) film, (b) T-LFO (110) film, (c) T-LFO (111) and (d) T-LFO (111)* films.

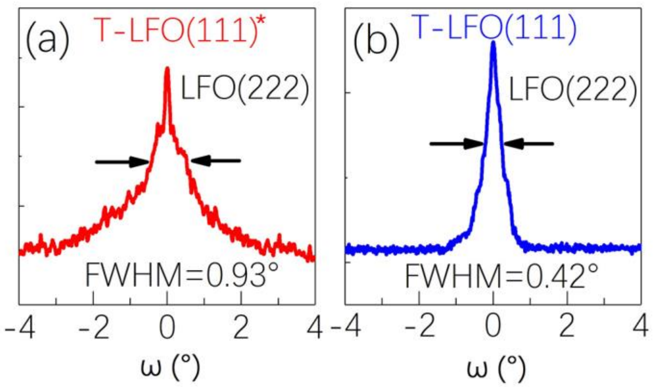

Figure S4. (a and b) XRD rocking curves of (a) T- LFO (111)* and (b) T-LFO (111) films, respectively.

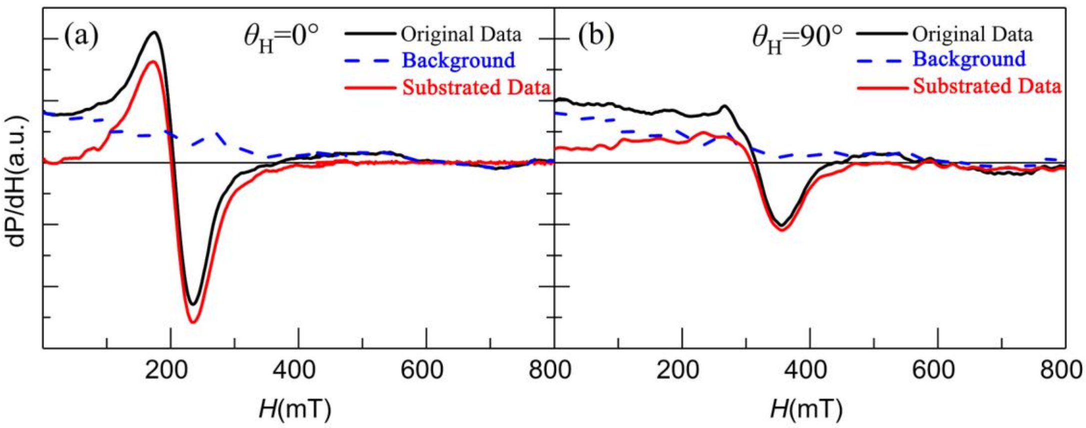

Figure S5. (a and b) FMR spectra of T-LFO (111)* films along (a) IP (θ_H=0°) and (b) OOP (θ_H=90°) orientations, respectively. The back solid lines, blue dash lines and the red solid lines represent the original signal, the signal from empty cavity and the signal that has subtracted the background, respectively.

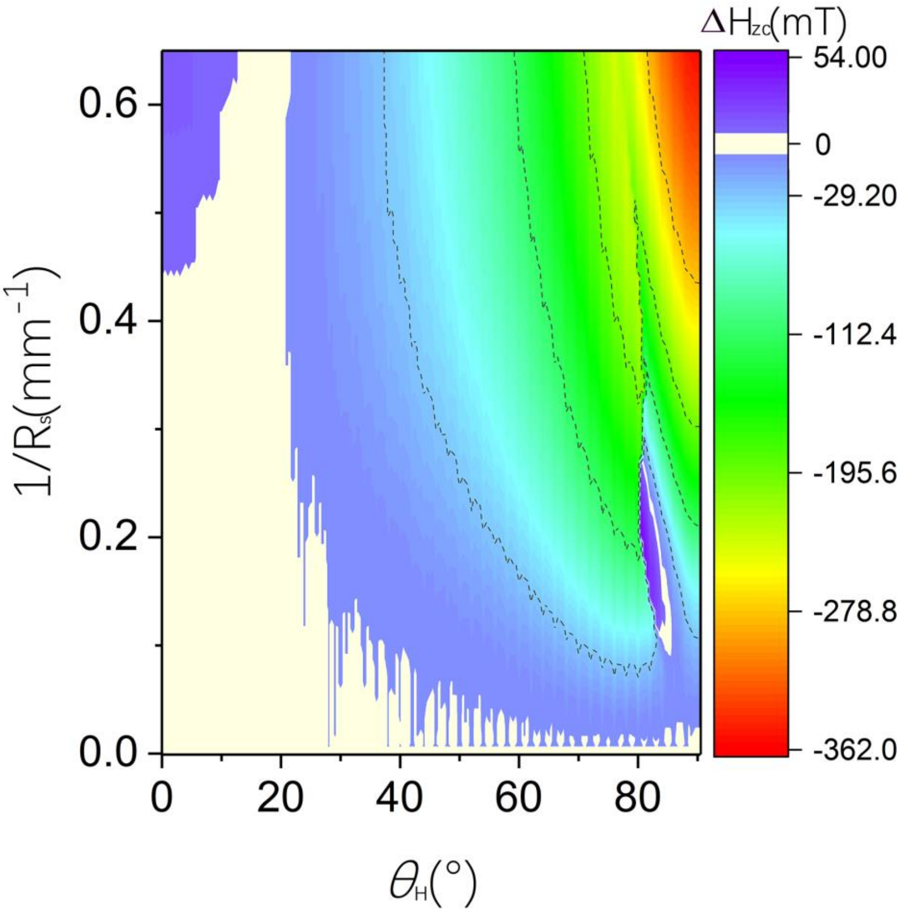

Figure S6. The simulated change of H_{zc} value (ΔH_{zc}) for T-LFO (001) films under bending (bending radius R_s) compared with that under the unbending state ($1/R_s$=0) at θ_H ranging from 0° to 90°.

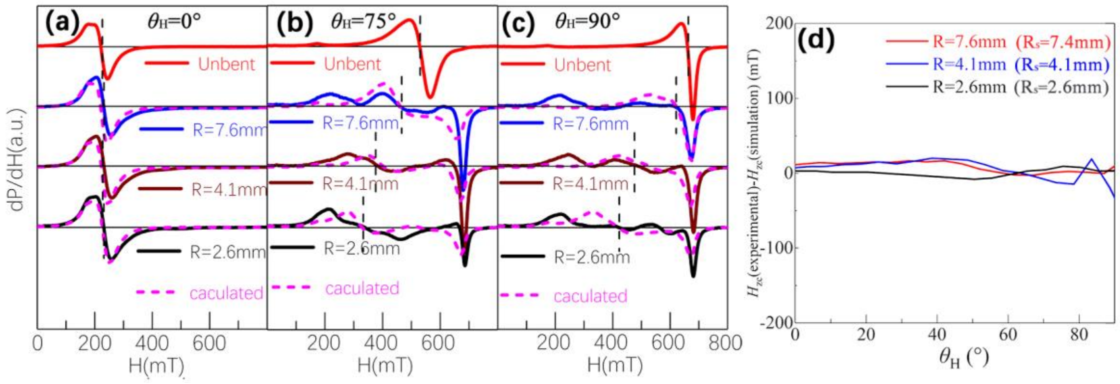

Figure S7. (a-c) Comparison of the FMR spectra for T-LFO films with different bending status at (a) θ_{H}=0°, (b) θ_{H}=75°, and (c) θ_{H}=90°. The purple-red dash lines represent the simulated (or calculated) FMR spectra. (d) The difference between experimental and simulated H_{zc}.

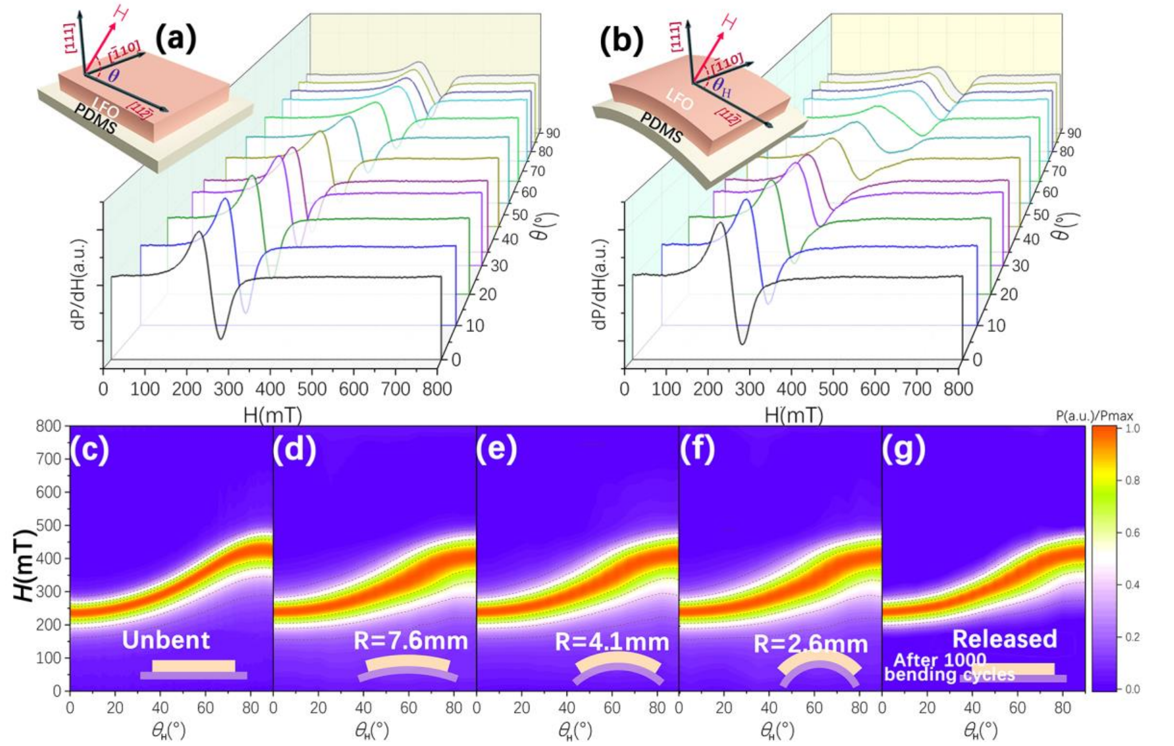

Figure S8. (a and b) Angular θ_{H} dependent FMR spectra for T-LFO (111) films with (a) unbending and (b) bending states. (c-g) Counter plot of the θ_{H} dependent integrated FMR spectra for the T-LFO (111) film with different bending status.

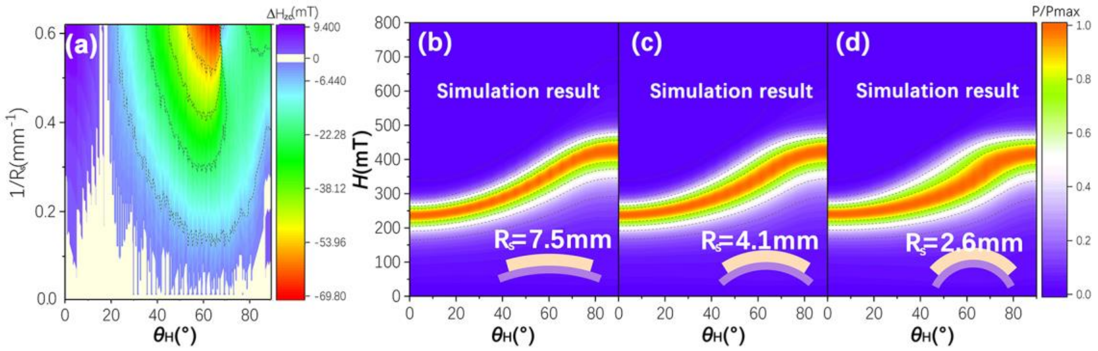

Figure S9. (a) The simulated change of H_{zc} value for T-LFO (111) films (ΔH_{zc}) under bending (bending radius R_s) compared with the unbending state ($1/R_s=0$) for θ_H ranging from 0° to 90°. (b-d) Counter plot of the simulated θ_H dependent integrated FMR spectra for the T-LFO (111) films with different bending status.

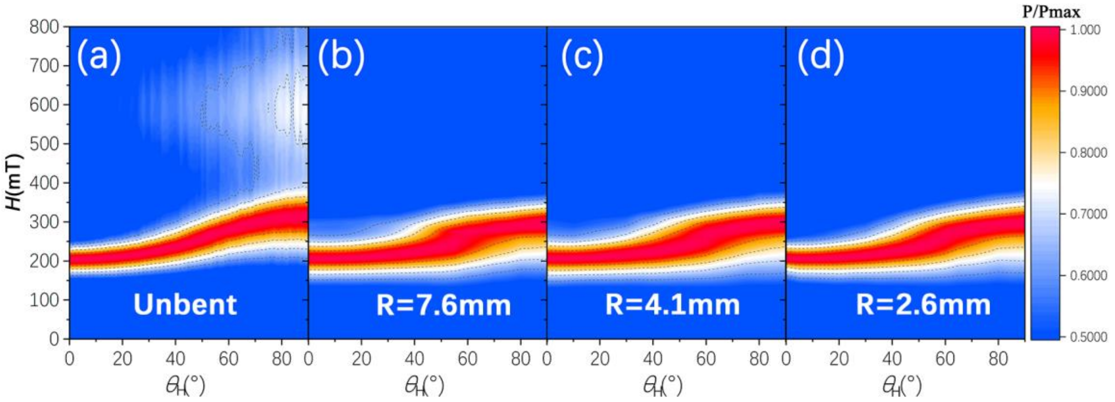

Figure S10. (a-d) Counter plot of the θ_H dependent integrated FMR spectra for the T-LFO (111)* film with bending states of (a) unbent, (b) R=7.5 mm, (c) R=4.0 mm, and (d) R=2.5 mm.

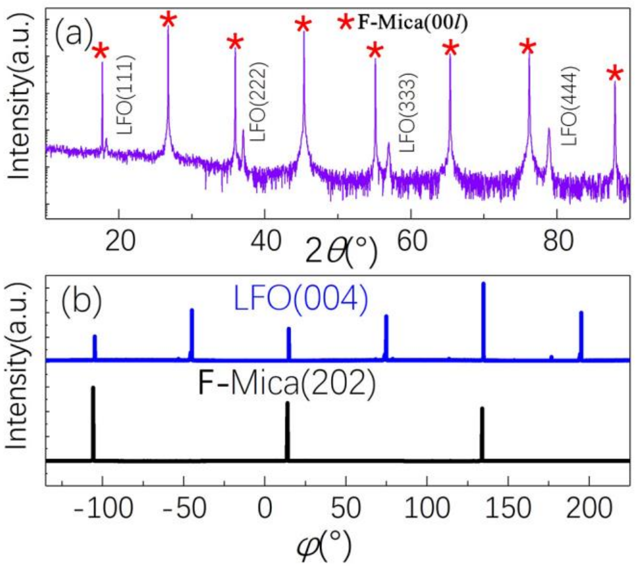

Figure S11. (a) Typical XRD θ-2θ scans for the NP-LFO sample with (111) OOP orientation. (b) φ-scan result for the NP-LFO (111) taken around from LFO (004) and F-Mica (202) peaks.

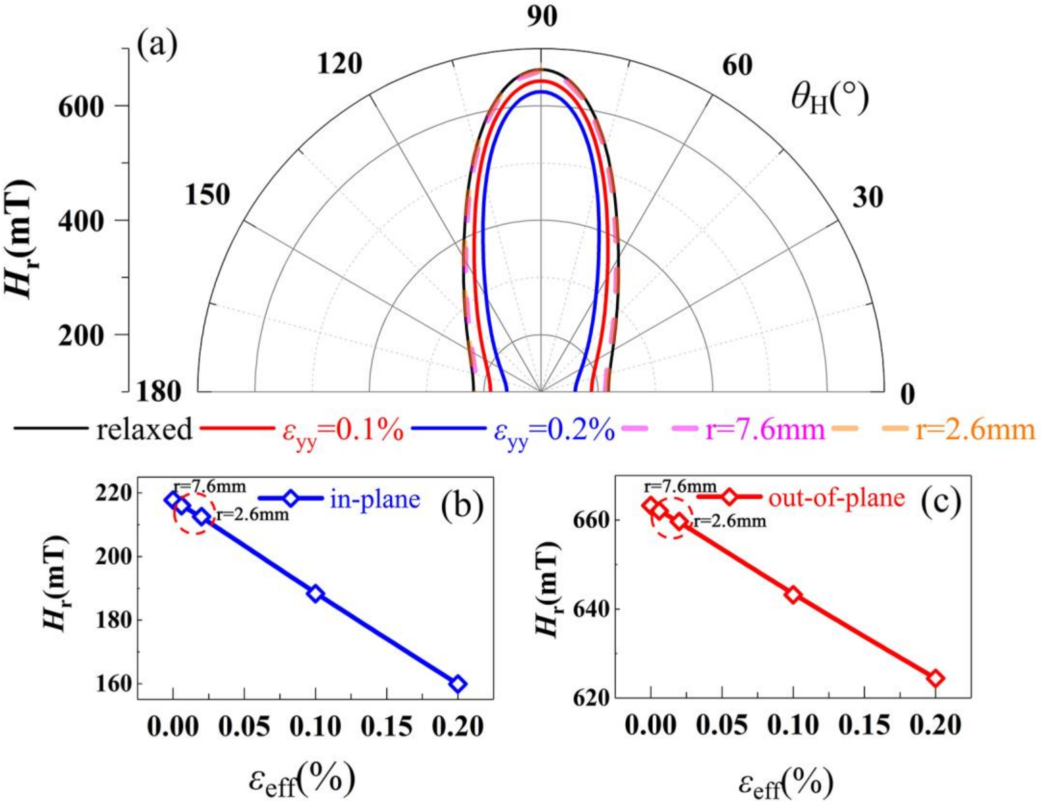

Figure S12. (a) Simulated angular θ_M dependent H_r for the T-LFO (001) film under different degrees of tensile strain along *y* axis. (b and c) the corresponding H_r along (b) in-plane and (c) out-of-plane directions under different degrees of tensile strain along *y* axis, respectively.

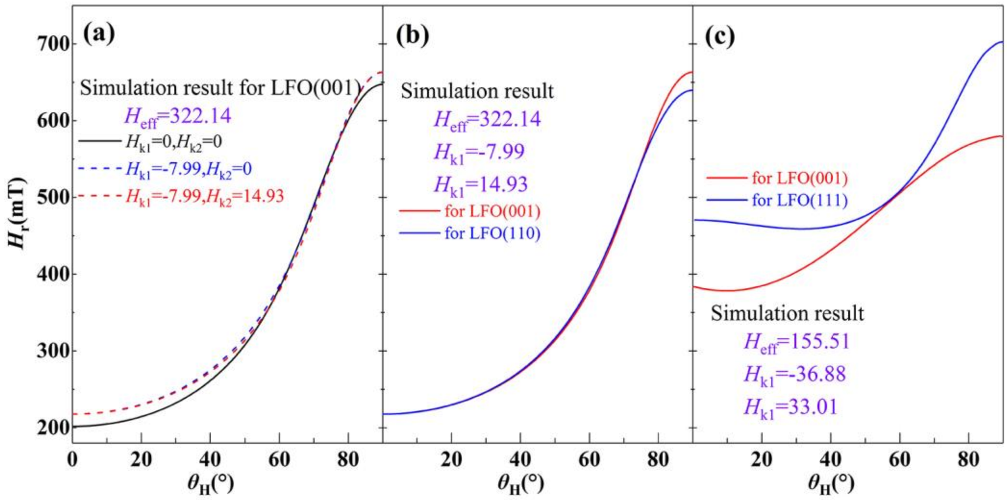

Figure S13. (a) Simulation lines for the angular θ_H dependent H_r that considering the contribution from H_{k1}, H_{K2} or not. (b and c) Simulation lines for the angular θ_H dependent H_r with the same parameters (for b, the parameters are from T-LFO (001) in Figure 1h; for c, the parameters are from T-LFO (111) in Figure 1h) for films with different OOP crystalline orientations.
